# Efficacy and Safety of Treatment with Plasma from COVID-19-Recovered Individuals

**DOI:** 10.3390/life13112184

**Published:** 2023-11-09

**Authors:** Mari Terada, Sho Saito, Satoshi Kutsuna, Noriko Kinoshita-Iwamoto, Tomiteru Togano, Akira Hangaishi, Katsuyuki Shiratori, Yuki Takamatsu, Kenji Maeda, Yukihito Ishizaka, Hiroshi Ohtsu, Masahiro Satake, Hiroaki Mitsuya, Norio Ohmagari

**Affiliations:** 1 Center for Clinical Sciences, National Center for Global Health and Medicine, Toyama, Shinjuku-ku, Tokyo 162-8655, Japan; 2 Disease Control and Prevention Center, National Center for Global Health and Medicine, Toyama, Shinjuku-ku, Tokyo 162-8655, Japankutsuna@hp-infect.med.osaka-u.ac.jp (S.K.);; 3 Department of Hematology, National Center for Global Health and Medicine, Toyama, Shinjuku-ku, Tokyo 162-8655, Japan; 4 Laboratory Testing Department, National Center for Global Health and Medicine, Toyama, Shinjuku-ku, Tokyo 162-8655, Japan; 5 Department of Refractory Viral Infections, National Center for Global Health and Medicine, Toyama, Shinjuku-ku, Tokyo 162-8655, Japan; 6 Division of Antiviral Therapy Joint Research Center for Human Retrovirus Infection, Kagoshima University, Sakuragaoka, Kagoshima 890-8544, Japan; 7 Department of Intractable Diseases, National Center for Global Health and Medicine, Toyama, Shinjuku-ku, Tokyo 162-8655, Japan; 8 Faculty of Health Data Science, Juntendo, Hongo, Bunkyo-ku, Tokyo 113-0033, Japan; 9 Central Blood Institute, Japanese Red Cross, Tatsumi, Koto-ku, Tokyo 135-8521, Japan

**Keywords:** COVID-19, SARS-CoV-2, convalescent plasma, antiviral therapy, emerging infectious diseases, neutralizing antibodies

## Abstract

Convalescent plasma therapy, which involves administering plasma from recovered coronavirus disease 2019 (COVID-19) patients to infected individuals, is being explored as a potential treatment for severe cases of COVID-19. This study aims to evaluate the efficacy and safety of convalescent plasma therapy in COVID-19 patients with moderate to severe illness. An open-label, single-arm intervention study was conducted without a control group. Plasma collected from recovered COVID-19 patients was administered to eligible participants. The primary endpoint was the proportion of patients who were placed on artificial ventilation or died within 14 days of transfusion. Secondary endpoints included clinical improvement, viral load measurements, and adverse event monitoring. A total of 59 cases were included in the study. The primary endpoint was evaluated by comparing the rate obtained in the study to an existing rate of 25%. The study also assessed clinical improvement, viral load changes, and safety endpoints through adverse event monitoring. Convalescent plasma therapy shows potential as a treatment option for COVID-19. This study aimed to provide evidence for the efficacy and safety of this therapy and may contribute to its future use in treating severe cases of COVID-19.

## 1. Introduction

The year 2019 marked the emergence of a formidable adversary, as a novel coronavirus called severe acute respiratory syndrome coronavirus 2 (SARS-CoV-2) unleashed a global pandemic we now know as coronavirus disease 2019 (COVID-19) [1,2,3,4]. Initially spread in Wuhan, Hubei Province, People’s Republic of China, at the end of December 2019, COVID-19 has since spread globally [1,2]. There have been 3,751,069 confirmed cases and 263,346 deaths worldwide (mortality rate: 7.0%), based on numbers up to early May 2020. In Japan, there are 15,462 reported cases at the time of this protocol preparation, with a fear of further outbreaks to come [1,5,6].

As the battle against this virulent adversary wages on, the medical community has marshaled an array of therapeutic candidates for treatment of COVID-19. However, further studies are necessary to determine how to treat severe cases. One treatment being explored, apart from antivirals, is the use of plasma from recovered patients, known as convalescent plasma therapy [2,3,4]. At its core, convalescent plasma therapy hinges on the simple yet powerful concept of transferring immunity. This can be used as a potential treatment for emerging and re-emerging infectious diseases that have no effective treatment options. In the recent past, this approach was used in patients with severe acute respiratory syndrome (SARS) and Middle East respiratory syndrome (MERS) [4,5,6,7,8,9,10]. The plasma from recovered patients contained neutralizing antibodies against SARS- and MERS-associated coronaviruses, which were believed to have antiviral effects when administered to patients presenting with those diseases. This treatment involves administering antibodies from the plasma of an individual who has recovered from an infectious disease to another patient dealing with the same disease and has been studied as a potential treatment for various infectious diseases [8,9,11]. Therefore, convalescent plasma therapy may be an effective treatment option for COVID-19 [7,8,10].

In cases of SARS-CoV-2 infection, it is now known that antibodies typically become positive in most patients within three weeks of illness onset. There have been 42 cases reported in China and South Korea where plasma collected from recovered patients was administered to severely ill patients, resulting in favorable outcomes [6,10,12]. A group of 15 patients in China who received convalescent plasma were initially considered to have a mortality rate of approximately 30%. However, following treatment, all of the patients survived, and no serious side effects were reported, indicating the potential of this treatment option [13]. In a report of 25 cases of convalescent plasma administration in the United States, plasma was collected and administered to patients without specific antibody titers, provided that the plasma donor was a recovered patient [7]. However, more deaths occurred in cases with low antibody titers, emphasizing the need for the appropriate evaluation of antibody titers and neutralizing activity to collect safe and highly effective plasma.

This study describes a protocol that will evaluate the efficacy and safety of convalescent plasma collected from individuals who have recovered from COVID-19. This trial was approved by the Certified Review Board of the National Center for Global Health and Medicine (Approval No.: NCGM-C-003607).

## 2. Materials and Methods

### 2.1. Experimental Design

This research was an uncontrolled, open-label, single-arm intervention study that solely focused on the administration of convalescent plasma. We abstained from employing randomization or blinding techniques. Given the ethical quandary surrounding the existence of a nontreatment group for COVID-19 patients diagnosed with moderate to severe illness, establishing a non-treatment group was considered ethically problematic. Furthermore, due to the prevailing circumstances during the enrolment period and other ethical considerations, the number of patients was limited; therefore, this research was conducted as a single-center study without a control group.

The convalescent plasma used in this study was previously collected and stored from a related convalescent plasma collection study (“Collection and Antibody Titer and Activity of COVID-19 Convalescent Plasma”, abbreviated as “COVIPLA-D”; ratified by the National Center for Global Health and Medicine Ethical Review Committee; Reference No. 3536). We evaluated the underlying factors [14] and neutralizing activity of IgG activity in earlier studies [15]. We also demonstrated the disappearance of severe acute respiratory syndrome coronavirus 2 (SARS-CoV-2) RNA (RNAemia) in a subset of 100 donors with varying degrees of disease severity [16]. Moreover, multiple studies have evaluated the efficacy, safety, disease progression, and utility of convalescent plasma therapy, ranging from small cohorts of patients to data of thousands of patients for experimental trials and systematic review [3,17,18,19,20,21]. These studies will aid in establishing the initial evidence regarding the usage of convalescent plasma in treatment. Several authors have also studied the risk of death and immunological aspects for this therapy, detailing the mechanism of action [4,22,23,24].

The intervention consisted of a single 200 mL dose of convalescent plasma. Dosage initiation commenced at a rate of 10 mL every 15 min via a dedicated administration route, which was subsequently escalated to an infusion rate of 100 mL per hour. The dosage was set in reference to a previous paper [25].

The study targeted patients diagnosed with COVID-19 with moderate to severe illness. A historical control group was deemed appropriate to compare and evaluate the efficacy of the treatment. The control group consisted of patients with similar disease severity who did not receive convalescent plasma. Based on comparisons of prognostic assessments, data from COVID-19 cases registered in the COVID-19 Registry Japan were matched by disease severity.

### 2.2. Target Population

The eligibility criteria for participants were defined based on ethical considerations and patient safety. Subjects to receive convalescent plasma were recruited from COVID-19 patients admitted to the implementing institution. An eligibility form comprising of the subject’s background information, such as their date of birth, gender, etc., and their subject identification code was kept on file to ensure that there were no problems with the selection and inclusion/exclusion criteria.

#### 2.2.1. Inclusion Criteria

Written informed consent was obtained from each participant or their legal representative. Participants were inpatients who had been diagnosed with SARS-CoV-2 infection by polymerase chain reaction, loop-mediated isothermal amplification, or other methods. Participants had to meet either of the following criteria at the time of hospitalization: (1) Saturation of peripheral oxygen value of 94% or lower; (2) Requirement of oxygen administration. Participants had to be 20 years of age or older at the time of enrollment.

#### 2.2.2. Exclusion Criteria

We excluded patients: (1) Pregnant or lactating; (2) With religious beliefs that did not permit the use of blood transfusions; (3) Already participating in an interventional study involving a therapeutic intervention for COVID-19; (4) Who had already received convalescent plasma; (5) With a history of allergy to blood products; (6) With plasma protein deficiencies, such as immunoglobulin A. Other patients judged inappropriate for inclusion in the study by the principal investigator or subinvestigator were excluded.

#### 2.2.3. Sample Size

The sample size for this study was 59 cases from the National Center for Global Health and Medicine. The study involved a safety evaluation; it included three interim analyses (plus the final analysis). The significance level was set to 5% (two-sided), and the power was set to 80%. Wu [25] reported that 25% of patients with non-mild disease required intubation. Similarly, a Japanese article reported similar results [26]. Therefore, assuming that the ratio of intubation in the existing treatment was 25% and expecting this ratio to be reduced to more than half (i.e., 12% or less), the number of cases was set using the O’Brien–Fleming type as the alpha spending function and the Pocock type as the beta spending function, and the sample size was calculated to be 59. The interim analysis was conducted targeting 15 cases in the first analysis, 30 cases in the second analysis, and 44 cases in the third analysis. In view of the irreversible nature of the treatment administered, it was assumed that there would be no dropouts during the course of the study, and 59 cases were determined to be the required number of patients. As our predetermined assumptions did not significantly change, the sample size in this study was not re-established. The SAS 9.4 proc seqdesign software (SAS Institute, Cary, NC, USA) was used for the sample size calculation.

#### 2.2.4. Spectrum of Disease (N = 44,415)

- Mild: 81% (36,160 cases). Definition: No pneumonia or mild pneumonia.
- Severe: 14% (6168 cases). Definition: Dyspnea, respiratory frequency ≥ 30/min, blood oxygen saturation ≤ 93%, partial pressure of arterial oxygen to fraction of inspired oxygen ratio < 300, and/or lung infiltrates > 50% within 24 to 48 h.
- Critical: 5% (2087 cases). Definition: Respiratory failure, septic shock, and/or multiple organ dysfunction.

### 2.3. Data Collection

Data collection for the clinical evaluation included clinical assessments, viral load measurements, blood tests, and adverse event monitoring. Observations and examinations were conducted and collected in accordance with the COVID-19 Registry (COVIREGI-JP) [27].

(1) Background information: Hospitalization date to day 1 of administration.

Date of birth, gender, race, smoking history, complications, nationality/race, pregnancy, background on COVID-19, overseas travel status, etc.

(2) Physical examination and findings during hospitalization: Hospitalization date, days 1, 3, 7, 14, (21), and 28 of administration.

Vital signs (temperature, blood pressure, pulse, respiratory rate, and oxygen saturation), height, weight, presence or absence of symptoms, oxygen administration, use of ventilator, examination for infection, etc.

On the day of administration (day 1), a detailed physical examination should be obtained 3 h after the start of transfusion.

(3) Blood tests: On the hospitalization date.

- Hemoglobin, hematocrit, white blood cell count, white blood cell fraction, platelet count, blood coagulation (APTT, PT-INR), albumin, AST, ALT, bilirubin, CRP, blood sugar, urea nitrogen, creatinine, LDH, creatine kinase, potassium, and sodium;
- Blood type and cross-matching (only on day 1 of administration);
- Infectious disease screening: HBsAg, HBsAb, HCVAb, HIV-1,2Ab, Syphilis-Ab, and HTLV-1,2-Ab (day of admission—day 1 of administration);
- Infectious disease screening: HIV 1 and 2, HCV core protein-HS, and HBV-DNA quantitative RT-PCR (day 90 of administration);

(4) Swab collection;

(5) Imaging diagnosis.

The process followed a well-defined timeline and procedure (described in the detailed procedure of Section 2.6) to ensure comprehensive and timely data collection.

Data containing personal information of subjects were erased during the transcription of electronic medical records to case report forms. Subsequently, a unique subject identification code was assigned. This identification code, commencing with “COVIPLA-R-01”, was structured without any discernible pattern in relation to the medical record ID. The last two digits were assigned chronologically in correspondence with the order of enrollment into the study.

In tandem with this process, an anonymization cross-referencing table, facilitating the identification of individual subjects, was created and stored. This table was accessible to the principal investigator, research collaborators, and monitoring personnel. To ensure the confidentiality and privacy of the subjects, the data management and analysis staff were not provided with access to anonymization tables. The supervisory staff were entrusted with the responsibility of carrying out data management tasks in a manner that precluded any possibility of identifying individual subjects. A further layer of assurance was provided by ensuring that all researchers and relevant personnel underwent ethics training, further affirming their commitment to ethical data handling practices.

External monitoring staff, as designated by the principal investigator, were granted access to records pertinent to the study, including case report forms, consent forms, and medical records. In such scenarios, the individual overseeing monitoring activities utilized a terminal with restricted access to the medical records. The usage of this terminal was conducted under the supervision of the principal investigator or an individual acting on behalf of the principal investigator.

Adequate safety management measures were implemented for the storage of study samples and related information throughout the duration of the study. Samples were securely stored in a locked deep freezer located at the National Center for Global Health and Medicine, with robust access controls in place to ensure data integrity. Paper-based information was subjected to stringent control measures; it was securely housed under lock and key within a cabinet situated in the office of the DCC Medical Director, which was accessible only through controlled entry. Electronic information was meticulously recorded on external electronic media, with the media itself subject to controlled access facilitated by a user ID and password system. Moreover, access to the information residing on the electronic media was rigorously regulated, following the same protocol as the paper-based information, and housed within the same locked cabinet. Access to these research data was strictly limited to researchers and collaborators affiliated with the research organization.

### 2.4. Data Analysis

Data analysis was conducted in this study with the intention-to-treat population serving as the primary analysis population. The primary endpoint was evaluated by calculating the proportion of patients on artificial ventilation management or who died within 14 days of transfusion. The rate obtained during the study was compared with the existing rate of 25%, which was reported in previous studies.

Secondary endpoints in this study encompassed a comprehensive array of evaluations aimed at garnering insights into the impact of convalescent plasma transfusion on patient outcomes. First and foremost, a meticulous assessment of clinical improvement was carried out at the 28-day mark post-transfusion, specifically in patients who received convalescent plasma transfusions. This evaluation entailed the calculation and subsequent assessment of percentages across seven predefined categories, affording a comprehensive overview of the therapeutic impact.

Another critical secondary endpoint involved the scrutiny of changes in SARS-CoV-2 viral load subsequent to convalescent plasma transfusion. Through a comparative analysis of SARS-CoV-2 viral load dynamics from pre-transfusion to day 14 of transfusion, as captured by nasopharyngeal swabs, a corresponding confidence interval was calculated. This approach furnished a quantifiable measure of the influence of convalescent plasma on viral load modulation. Furthermore, the evaluation of symptom shortening hinged on the analysis of trends exhibited by blood test data over a span of time.

Due to the potential for a shift in treatment approach or an enhancement in mortality rates within the cohort of patients with mild disease, recalibration of the study’s participant count was a possible course of action. This recalibration could be executed by drawing upon existing information at the juncture of the interim analysis, provided that substantial deviations from the initially projected assumptions became evident. For such instances, meticulous records detailing the conclusions arrived at were maintained, and authorization was sought from the Specified Clinical Research Review Committee to initiate the process of re-evaluation.

A sub-group analysis was not planned due to the small sample size (maximum of 59 cases). Interim analysis was conducted after 15, 30, and 44 cases were evaluated, with safety and efficacy being the focus of the analysis. Adverse events that could affect the continuation of the study were identified, and both effective and ineffective discontinuations were evaluated based on dilution criteria. Statistical analysis was conducted using the SAS 9.4 proc seqdesign software, with a significance level of 5% (two-sided) and 80% power. Confidence intervals of 95% were also calculated for the primary endpoint.

### 2.5. Ethical Considerations

Ethical considerations were taken into account for the study, particularly with regard to informed consent, institutional review board approval and monitoring, and safety monitoring and reporting procedures.

The informed consent process and procedures were carefully followed. Prior to participation in the study, the COVID-19 patients selected for the study intervention, or their legal representatives, were given an explanation of the nature of the study in terms that were easily understood, using the consent explanation document approved by the Clinical Research Review Committee. Sufficient time was provided to answer questions and discuss participation in the study. It was emphasized that consent was freely given and that the subject/proxy would not be treated adversely if they did not consent. The patient was allowed to withdraw consent at any time before plasma transfusion without any adverse treatment. If the patient withdrew consent after administration, the data was not used for analysis, but the patient was asked to cooperate with any necessary observations to confirm safety.

In cases where the subject had a diminished level of consciousness or cognitive decline, informed consent was obtained from the subject’s legal representative. The subject proxies were selected from the subject’s close relatives. When the subject regained consciousness and was deemed fully capable of understanding, their consent was also obtained before plasma transfusion.

When obtaining consent for study participation, the signatures of the investigator who provided the briefing and the subject/proxy who received the briefing, the date of the briefing, and the date consent was given were noted on the consent form. A copy of the briefing and consent form was given to the participant, and the original consent form was kept at the study site. Any procedures related to the study were implemented after obtaining the necessary consent for said procedures. Consent was obtained electronically or orally, as appropriate, in light of the risk of infection due to contamination of the consent document, and the fact that potential legal representatives were also persons in close contact with the patient and had difficulty leaving the house. Written consent was obtained as soon as possible, at a stage when written consent could be obtained.

Institutional review board approval and monitoring were obtained before the study commenced, and the study was monitored throughout its duration. Safety monitoring and reporting procedures were put in place to ensure the safety of the study participants. Any adverse events were promptly reported to the institutional review board and relevant authorities.

### 2.6. Detailed Procedure

The timeline and actions in the COVIPLA-R study were carefully outlined to ensure a systematic approach. A comprehensive overview of the study’s timeline and corresponding actions can be found in Table 1, providing details such as admission dates, days of plasma administration, medical examinations, laboratory tests, and documentation of adverse events.

The enrollment period concluded upon reaching the designated count of patients receiving convalescent plasma. Considering the necessity to match blood types for plasma transfusion, candidates with compatible blood types were identified just before the target patient number was achieved. These candidates received thorough briefings to obtain their informed consent.

Following the enrollment period, the observation phase came to an end when all subjects had completed their 3-month post-dose visits and successfully attended all scheduled hospital appointments.

In the clinical trial, the participation of a patient was suspended if any of the following criteria were met: (1) The subject withdrew consent; (2) The investigators determined that the risks of participation outweighed the benefits. However, if a subject had already been transfused with convalescent plasma, the subject was continued to be observed for adverse events for safety reasons. The suspension criteria were set with ethical considerations based on the perspective of subject safety.

If transfusion was discontinued without completing the 200 mL of plasma transfusion due to adverse effects after convalescent plasma transfusion, necessary medical care and follow-up were provided for 24 h after the transfusion was discontinued. This was reported as an adverse event/serious adverse event. Furthermore, as stipulated in the study protocol, screening for infections was performed 90 days after the discontinuation of transfusion. Any remaining plasma was collected and used for research purposes, as specified in the plasma collection study (COVIPLA-D: NCGM-003536).

In the event of suspension of the study, the Efficacy and Safety Evaluation Committee would deliberate on the appropriateness of continuing the study. The study would be temporarily suspended if any of the following situations occurred: (1) The risks of participation outweighed the benefits, based on the results of the interim analysis or other factors; (2) Serious noncompliance with applicable laws and regulations of the research protocol was discovered; (3) The Authorized Clinical Research Review Committee expressed an opinion; (4) The Minister of Health, Labor and Welfare requested or recommended discontinuation; (5) The Efficacy and Safety Evaluation Committee decided to suspend the study. If the Efficacy and Safety Evaluation Committee determined after deliberation that there was no reason not to continue the study, subject enrollment would resume. If discontinuation of the study was deemed appropriate, the principal investigators at all medical institutions would be promptly notified, and the director of the study sites and the Authorized Clinical Research Review Committee would also be notified. Additionally, the subjects would be contacted to arrange their next visit and enter the post-investigation period.

The study was terminated when all of the following items were completed: (1) Enrollment of subjects in the study and completion of the observation period; (2) Preparation of the primary endpoint report, clinical study report, and summary of the clinical study report; (3) Submission of the primary endpoint report to the Minister of Health, Labor and Welfare; (4) Submission of a summary of the clinical study report, study protocol, and statistical analysis plan to the Minister of Health, Labor and Welfare; (5) Submission of the primary endpoint report, clinical study report, and summary of the clinical study report to the directors of each study site; (6) Registration of the summary of the study results on the Japan Registry of Clinical Trials; (7) Reporting of the publication of study results to the directors of each study site.

## 3. Expected Results

The use of convalescent plasma therapy has shown potential as a treatment option for COVID-19, with several studies reporting promising results. However, it is important to consider the potential risks associated with this therapy, especially because the plasma is collected from recovered COVID-19 patients and may contain unknown infectious agents.

To minimize these risks, several measures were proposed, including performing viral testing and cross-matching, conducting human leukocyte antigen (HLA) antibody testing, and closely monitoring patients during and after the transfusion. Additionally, we emphasized the importance of obtaining informed consent from patients and providing detailed information about the potential risks and benefits of the treatment.

Overall, a comprehensive plan was prepared for the safe and ethical use of convalescent plasma therapy in COVID-19 patients, with the potential to provide valuable evidence for the efficacy of this treatment option.

### 3.1. Expected Benefits

Participation in this study offered patients the opportunity to receive convalescent plasma therapy, which has the potential to improve respiratory symptoms and prevent the progression of COVID-19 to severe illness. If the study findings demonstrate the efficacy of convalescent plasma therapy, this may provide important evidence for the use of this treatment option in the future, benefiting society at large.

### 3.2. Anticipated Disadvantages

Convalescent plasma, the focus of this study, undergoes testing, processing, and storage in alignment with the protocols for plasma obtained from component blood donations. As a result, it may present comparable risks to those associated with adverse reactions linked to fresh-frozen plasma. In scenarios where the plasma donor carries an undetected infectious disease, particularly those challenging to screen for at the present juncture, there exists a potential hazard for the recipient of the administered plasma to contract said infectious disease.

In addition to the above concerns, the spectrum of potential adverse events encompasses hypersensitivity, shock, anaphylaxis, and multi-organ failure, which may transpire due to immune responses or antigen–antibody reactions. Furthermore, HLA antibodies present in blood products may pose a risk of acquiring transfusion-related acute lung injury (TRALI) after plasma administration.

### 3.3. Methods to Minimize Risk

To alleviate the potential for adverse reactions attributed to infection or immune responses, we performed identical viral testing and cross-matching procedures as those employed for regular blood donations. Furthermore, the blood was administered within a medical setting under constant supervision by healthcare professionals, who diligently monitored any changes in the recipients’ physical condition.

To attenuate the risk of TRALI during administration, HLA antibody testing was conducted during plasma collection. This approach was particularly applied to cases involving transplant recipients and individuals with a history of prior transfusions, aiming to validate outcomes and bolster the procedure’s safety. To ensure a well-informed decision-making process, patients selected for plasma transfusion received comprehensive explanations regarding the potential benefits and risks linked to convalescent plasma transfusion. Notably, a comprehensive breakdown of the associated risks was provided during the process of securing subject consent.

As part of the comprehensive follow-up protocol, we planned post-transfusion infectious disease assessments at the three-month juncture post-transfusion. This comprised the quantification of HBV-DNA, evaluation of HCV core protein, and HIV (1 and 2) antibody tests. These measures were implemented to mitigate potential risks and ensure the utmost safety and well-being of the patients.

## Figures and Tables

**Table 1:** Timeline and actions in the COVIPLA-R Study.

Items	Admission Date	Day 1 of Administration		Day 3 of Administration	Day 7 of Administration	Day 14 of Administration	Day 21 of Administration	Day 28 of Administration	Day 90 of Administration	Suspension of Administration
		Pre-Administration	Post-Administration							
**Allowance**	**−3**	**―**		**±1**	**±1**	**±3**	**±3**	**±3**	**+14**	**―**
**COVIPLA-R Study Count**	**―**	**D1**		**D3**	**D7**	**D14**	**D21**	**D28**	**D90**	**―**
Informed consent	X									
Eligibility confirmation	X									
Plasma administration		X								
Basic information on admission/travel information	X									
Comorbidities, signs and symptoms, and pathogen testing	X									
Admission history (including vital signs)	X	X	Xg	X	X	X	(X)	X		Xh
Medical examination, interview, and physical examination	X	X	Xg	X	X	X	X	X		Xh
Swab collection	X			X	X	X	(X)	X		
Blood test (biochemistry and blood count)	X			X	X	X	(X)	(X)		X
Blood test (blood type and cross-matching)		X								
Blood test (infectious disease screening)	Xe								Xe	
Urine tests (urinalysis)	X									
Radiology (chest X-ray and/or CT)	X			X	X	X	(X)	(X)		
Confirmation of concomitant medications		X		X	X	X	X	X		
Confirmation of adverse events			X	X	X	X	X	X	Xd	X
Phone contact							Xf			

X: Required. (X): Required for continued hospitalization, otherwise at physician’s discretion. d: Serious adverse events only. e: Only events based on infectious disease screening (immunodeficiency virus 1 and 2, hepatitis C virus core protein–heparan sulfate, hepatitis B virus-deoxyribonucleic acid quantitative reverse transcription polymerase chain reaction). f: If the patient was discharged from the hospital, examination via telephone was required. g: Follow-up 3 h after initiation of treatment, including observation of adverse events. h: 24-h follow-up. CT: computed tomography.

## Data Availability

No new data were created or analyzed in this study. Data sharing is not applicable to this article.
